# The Guillotine

**Published:** 1875-04

**Authors:** 


### THE GUILLOTINE.

As is well known, the mode of taking the lives of doomed persons in France is by decapitation with a heavy knife, descending in a groove. Recent executions in Paris have called forth a vehement discussion, and the severe strictures of a portion of the press upon this mode of terminating human life. It is denounced as brutal in the extreme. It is asserted that by severing the head from the trunk, ordinary sensation is lost, while hearing, sight, and smell, with the whole apparatus of consciousness and intellect, remain active for some hours. It has been maintained that the trunk dies quietly and painlessly, in a few minutes; while the brain, shielded by atmospheric pressure, retains its blood, and, consequently, its life, for not less than three hours.

This is the old story, so often exploded. It was told of Charlotte Corday that her cheeks blushed at the insult offered her by her executioner, who lifted her decapitated head high in air, and smote it upon the cheek.

All this is absurd. The operation of the great knife is as painless as the scratching of a finger. It is over in a few seconds—perhaps in a single second, so far as conscious suffering is concerned. This is made evident by the fact that while the brain possesses a considerable quantity of blood after decapitation, the blood rapidly becomes venous for want of oxygen, the condition being like that in complete asphyxia, in which consciousness vanishes in ninety seconds. The physical shock sustained from the guillotine would of itself, moreover, paralyze all nervous function too completely to admit of consciousness taking place during the brief interval necessary for the thorough deoxidation of the blood in the brain. Beyond the momentary impact of the descending knife on the throat, no further sensation can be felt. Indeed, this mode of execution is so painless as almost to weaken the deterrent force of capital punishment; certainly to deprive those who would abolish that punishment of any argument deducible from the "horrors of the guillotine."

---

### SUNSHINE AND SLEEP.

No syrup of poppies, no tincture of opium, no powders of morphine, can compare in sleep-inducing power with sunshine. Let sleepless people court the sun. The very worst soporific is laudanum, and the very best is sunshine. Therefore, it is very plain that poor sleepers should pass as many hours of the day in sunshine, and as few as possible in the shade. Many women are martyrs, and do not know it. They shut the sunshine out of their houses and hearts, they wear veils, they carry parasols, they do all that is possible to keep off the subtlest and yet most potent influence which is intended to give them strength, and beauty, and cheerfulness. Is it not time to change all this, and so get roses and color in your pale cheeks, strength in your weak backs, and courage in your timid souls? The women of America are pale and delicate; they may be blooming and strong, and the sunlight will be a potent influence in this transformation.

---

The expression of truth is simplicity.

